# Advanced Vaccine Design Strategies against SARS-CoV-2 and Emerging Variants

**DOI:** 10.3390/bioengineering10020148

**Published:** 2023-01-22

**Authors:** Jianzhong Zhang, Yutian Xia, Xuan Liu, Gang Liu

**Affiliations:** 1 Center for Molecular Imaging and Translational Medicine, State Key Laboratory of Molecular Vaccinology and Molecular Diagnostics, School of Public Health, Xiamen University, Xiamen 361102, China; 2 Innovation Center for Cell Biology, State Key Laboratory of Cellular Stress Biology, School of Life Sciences, Xiamen University, Xiamen 361102, China

**Keywords:** vaccine design, SARS-CoV-2, COVID-19, variants

## Abstract

Vaccination is the most cost-effective means in the fight against infectious diseases. Various kinds of vaccines have been developed since the outbreak of COVID-19, some of which have been approved for clinical application. Though vaccines available achieved partial success in protecting vaccinated subjects from infection or hospitalization, numerous efforts are still needed to end the global pandemic, especially in the case of emerging new variants. Safe and efficient vaccines are the key elements to stop the pandemic from attacking the world now; novel and evolving vaccine technologies are urged in the course of fighting (re)-emerging infectious diseases. Advances in biotechnology offered the progress of vaccinology in the past few years, and lots of innovative approaches have been applied to the vaccine design during the ongoing pandemic. In this review, we summarize the state-of-the-art vaccine strategies involved in controlling the transmission of SARS-CoV-2 and its variants. In addition, challenges and future directions for rational vaccine design are discussed.

## 1. Introduction

The prevention and control of infectious diseases is the leading challenge to human progress and survival, similar to the ongoing pandemic that poses an unprecedented crisis to the world. Controlling the source of infection (isolation or quarantine), cutting off the transmission route (wearing masks, social distancing), and protecting susceptible populations (such as vaccination), are three major weapons fighting against infectious diseases practically in public health [1]. Vaccines, the greatest invention in medical history, could make vaccinated subjects less susceptible by eliciting an active immune response [2]. Vaccination has been proven a cost-effective public health intervention in history and protected millions of people exempt from various diseases [3,4]. Thanks to past experiences in vaccine design and manufacturing, the pipeline for severe acute respiratory syndrome coronavirus 2 (SARS-CoV-2) was rapidly established to tackle this outbreak situation [5,6]. Lessons learned from severe acute respiratory syndrome coronavirus (SARS-CoV) [7], Middle East respiratory syndrome coronavirus (MERS-CoV) [8], and influenza virus [9], etc. made researchers design vaccines empirically, which shorted trial and error time while an effective SARS-CoV-2 vaccine was urgently important. Additionally, the pandemic boosted the development of vaccine technologies, and an increasing number of novel strategies were exploited to meet the urgent need for effective vaccines.

In the hurdle against the pandemic, various kinds of vaccines were prepared and tested for efficacy against coronavirus disease 2019 (COVID-19), including whole inactivated virus vaccines [10], live-attenuated virus vaccines [11,12], subunit vaccines [13,14,15], viral vector vaccines [16,17,18], DNA vaccines [19], and mRNA vaccines [20,21], etc. As of 13 December 2022, 175 vaccines have entered clinical trials, and over 199 vaccine candidates are in the preclinical stage [22]. Among these, mRNA vaccines represented by BNT162b2 (Pfizer-BioNTech) [20] and mRNA-1273 (Moderna) [21] have drawn considerable attention as a revolutionary innovation in controlling the spread of SARS-CoV-2. Produced by in vitro transcription, mRNA can encode target proteins in the cytoplasm, thus reducing the risk of integrating into the genome compared with DNA vaccines. Furthermore, mRNA vaccines can induce both humoral and cellular immunity with high efficiency, which play a critical role in defending against virus infection. Despite significant protective efficacy against COVID-19 being reached (BNT162b2 conferred 95% protection [20], the mRNA-1273 vaccine showed 94.1% efficacy [21]), possible immune evasion may occur owing to newly emerging variants. Some emergent new variants of concern (VOCs) [23,24], such as Alpha (B.1.1.7), Beta (B.1.351), Gamma (P.1), Delta (B.1.617.2), and Omicron (B.1.1.529), have demonstrated increased transmissibility, infectivity, hospitalization, and mortality. These mutated strains bring new challenges to preventing and controlling COVID-19 and decreased protective efficacy of existing vaccines has been reported [25,26]. Vaccines that provide more effective and broad-spectrum protection are required.

**Scheme 1:**
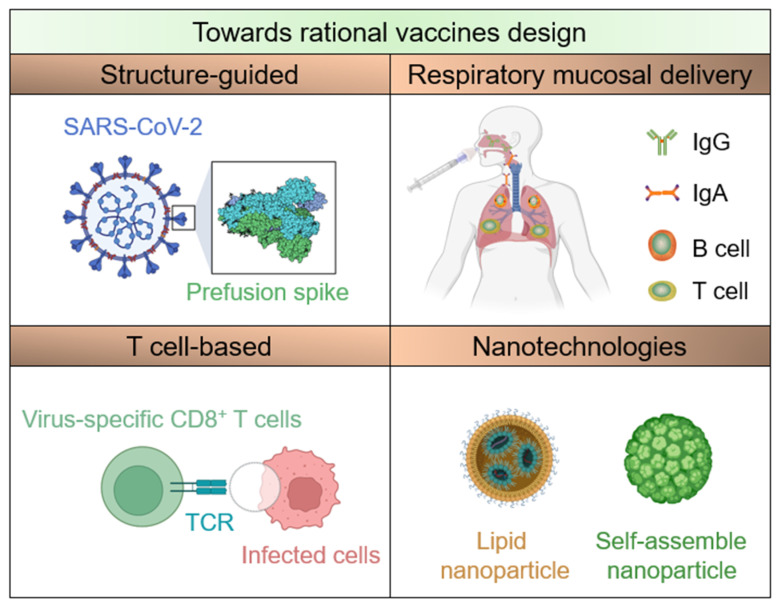
Insights towards rational vaccine design. Typical approaches involved in the COVID-19 vaccine candidates are discussed, including structure-guided vaccine design, T-cell-based vaccines, respiratory mucosal delivery, and enabled nanotechnologies. A schematic diagram is involved in illustrating each strategy.

With a more profound knowledge of the immune system, besides mRNA vaccines, some emerging concepts and advancing technologies are considered to address the widespread public concerns about vaccine effectiveness against COVID-19. Following the above regard, circular RNA vaccines [27], chimeric protein-based vaccines [28,29,30,31], virus vector-based vaccines [32,33], and nanoparticle vaccines [34,35], etc., are potential powerful candidates combating COVID-19. In fact, these vaccines embody the wisdom of optimized design principles compared with empirically prepared candidates. To advance vaccinology and provide some insights into this field, we summarize the state-of-the-art novel vaccine design approaches in this review. Here we highlight the role of structure-guided vaccine design [36,37], T-cell-based vaccines [38,39,40], respiratory mucosal delivery [41,42,43], and enabled nanotechnologies [44,45] (Scheme 1).

## 2. Overview of SARS-CoV-2 and Its Variants

**Figure 1:**
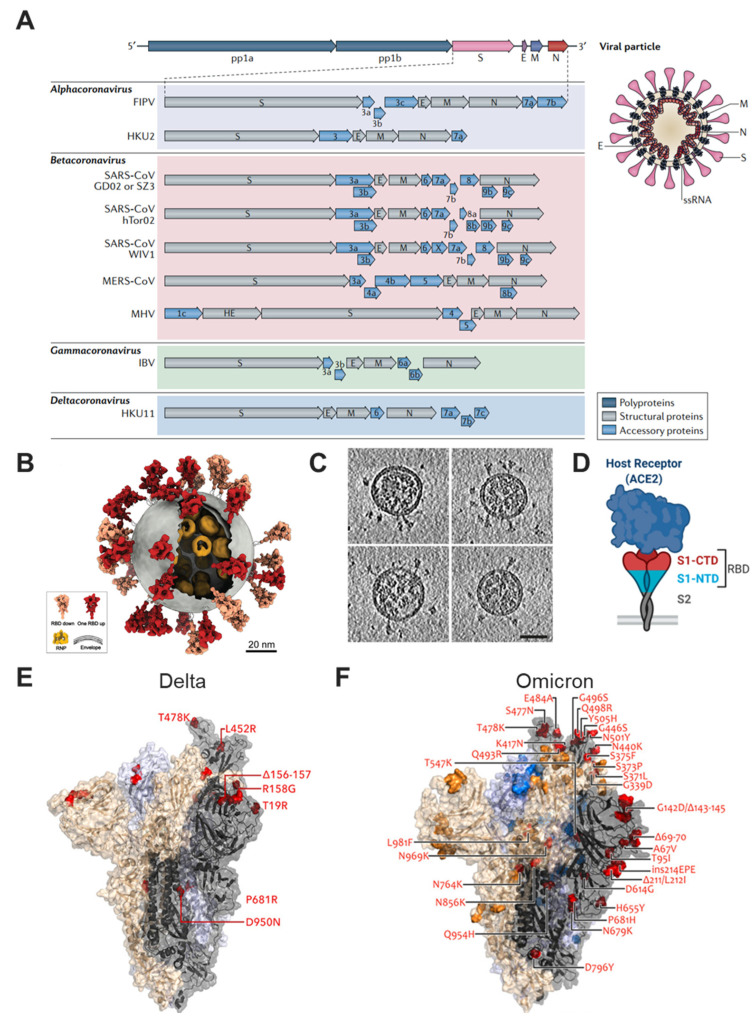
Overview of SARS-CoV-2 and its variants. (**A**) The genomes, genes and proteins of different coronaviruses. They are mainly constituted by positive-sense, single-stranded RNA (ssRNA), envelope glycoproteins spike (S), envelope (E), membrane (M) and nucleocapsid (N). Feline infectious peritonitis virus, FIPV. Middle East respiratory syndrome coronavirus, MERS-CoV. Mouse hepatitis virus, MHV. Infectious bronchitis virus, IBV. Reproduced with permission from Ref. [46]. Copyright 2018, Springer Nature. (**B**) The molecular architecture of the SARS-CoV-2 Virus. The RBD of spike protein exists “up” (red) and “down” (salmon) conformation. Ribonucleoprotein, RNP. Reproduced with permission from Ref. [50]. Copyright 2020, Elsevier. (**C**) Four representative tomographic slices of SARS-CoV-2 virions. Scale bar 50 nm. Reproduced with permission from Ref. [51]. Copyright 2020, Springer Nature. (**D**) S protein targets ACE2 through RBD in S1 subunit. RBD is the receptor-binding domain, and S1-CTD and S1-NTD are the C-terminal and N-terminal domains of S1. Reproduced with permission from Ref. [54]. Copyright 2020, MDPI. (**E**) Delta and (**F**) Omicron variants of SARS-CoV-2. Representative mutations in the spike protein are presented. Reproduced with permission from Ref. [66]. Copyright 2021, Wiley.

SARS-CoV-2 is another coronavirus that could cause fatal respiratory illness in humans since the outbreak of SARS-CoV and MERS-CoV at the beginning of the 21st century. Same as the other betacoronaviruses [46], SARS-CoV-2 is a positive-sense and single-stranded RNA virus, its genome sequences are almost identical and share 79.6% sequence identity with SARS-CoV [47] (Figure 1A). Among the encoded structural proteins (membrane (M) protein, nucleocapsid (N) protein, Envelope (E) protein, and spike (S) protein) [48,49], the envelope glycoprotein spike (S) confers SARS-CoV-2 crown-like appearance (corona) [50,51] (Figure 1B,C), and more critically, the spike is a protein that binds to the angiotensin-converting enzyme 2 (ACE2) receptor of host cells and mediates viral entry [52,53,54] (Figure 1D).

The transmembrane spike glycoprotein contains two subunits, S1 (surface ectodomain) and S2 (transmembrane domain); the receptor binding domain (RBD) of S1 is responsible for viral attachment via ACE2, and then S2 mediates the fusion process of viral and host membranes [55,56]. As an important target for neutralizing antibodies (nAbs) [57,58] and also for T-cell responses [59], RBD or full-length S protein becomes the main target antigen of choice [60]. However, high rates of gene mutations in the viral spike protein raised concerns that emerging variants might lead to re-infection or a new wave of outbreak. Some mutations (D614G, N501Y, etc.) could increase the tightness and affinity of binding with the ACE2 receptor, thus increasing the infectivity of SARS-CoV-2 variants [61,62], and evading the immune response elicited by natural infection or administrated vaccines supposed for wild-type viruses [63,64]. Following the Delta variant, the Omicron variant, which contains at least 32 mutations in the spike protein and escapes the majority of existing SARS-CoV-2 nAbs [65], has become the dominant strain in many countries worldwide [24,66,67] (Figure 1E,F). Given the massive mutations in spike protein, a more contagious variant than any other VOCs found so far may occur in the near future, collaboration from interdisciplinary communication and scientific understanding of the emerging "X variant" is required. Under the circumstance that administered vaccines are mismatched with circulating variants, traditional formulations such as inactivated vaccines hardly provide cross-protection, and advanced vaccine technologies are needed. The four novel vaccine design strategies (Table 1) are discussed below, and we compared their advantages and limits (Table 2).

## 3. Structure-Guided Vaccine Design

From the first to second to third generation vaccines, rational design rather than empiricism is getting recognized in modern vaccinology. Progress in structural biology has contributed greatly to the rational vaccine design in recent years [36,81], which provides an on-the-shelf technique for the fight against emerging infectious diseases.

The spike protein of SARS-CoV-2 is a homo-trimer [37], just such as some other class I viral glycoproteins of enveloped RNA viruses such as HIV [82,83] and influenza [84,85], etc. Generally, sufficient neutralizing antibodies in serum or mucosal secretions induced by vaccines play a vital role in blocking viruses. To expose neutralization-sensitive epitopes to B cells, the prefusion-stabilized spike proteins are preferred, where enhanced antigen homogeneity and stability are featured [86]. Following this principle, until now, some approaches have been developed to keep the native-like prefusion conformation, and improved immune responses were found as compared to the wild-type spike glycoproteins [68,71,87].

**Figure 2:**
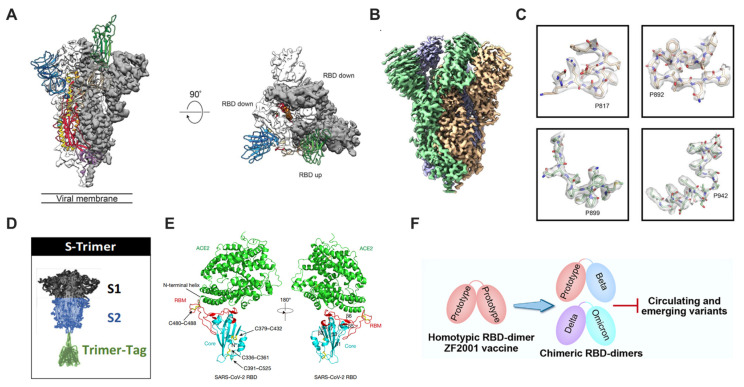
Structure-guided vaccine design. (**A**) Structure of SARS-CoV-2 spike protein in the prefusion conformation. The "up" conformation is the receptor-accessible state, and the "down" conformation corresponds to the receptor-inaccessible state. Reproduced with permission from Ref. [56]. Copyright 2020, AAAS. (**B**) The electron microscope density map of trimeric HexaPro and (**C**) Four proline substitutions (F817P, A892P, A899P and A942P) unique to HexaPro. Based upon general protein stability principles, 100 structure-guided spike designs were characterized, and 26 individual substitutions can increase protein yields and stability. Through the combination of beneficial substitutions, HexaPro with six proline substitutions was chosen to owe to its higher expression level and heat stability. Reproduced with permission from Ref. [37]. Copyright 2020, AAAS. (**D**) Illustration of S-Trimer with homo-trimeric prefusion conformation. Briefly, the spike protein was fused with the human C-propeptide of α1(I) collagen, which is capable of self-trimerization via disulfide bonds. Reproduced with permission from Ref. [71]. Copyright 2020, Springer Nature. (**E**) The overall structure of the SARS-CoV-2 RBD bound to ACE2. RBD core is in cyan, RBM is in red, and ACE2 is in green. Reproduced with permission from Ref. [100]. Copyright 2020, Springer Nature. (**F**) Schematic diagram of RBD-dimer. Homotypic or chimeric dimers were constructed to elicit broader protection against emerging variants. Reproduced with permission from Ref. [30]. Copyright 2022, Elsevier.

The most widely used strategy is proline mutation [88], which influenced the design of the vaccine critically during the ongoing pandemic. Encouragingly, the S-2P strategy (comprising proline substitutions) is broadly applicable and could be regarded as a universal manner in different coronaviruses [89,90]. As a key target antigen for vaccine development, the spike protein of coronaviruses is in a metastable prefusion conformation, and maintaining its neutralization-sensitive epitopes is indispensable to improve antigenicity and protein expression. Under the guidance of the protein-engineering strategy, two consecutive proline substitutions in the S2 subunit were screened, and it was determined that this construct could promote homogeneous preparations of prefusion spikes. For the spike protein of SARS-CoV-2, its Cryo-EM structure in the prefusion conformation was rapidly resolved within one month, which was assisted by the S-2P strategy (proline substitutions at residues K986 and V987) to obtain a high titer of prefusion-stabilized S ectodomain [56] (Figure 2A). The atomic-level structural information has guided and accelerated the vaccine’s design and development, including mRNA vaccines [21,68], adenovirus vector-based vaccines [91,92], recombinant protein vaccines [93,94,95], etc. Upon the basis S-2P, S-6P (HexaPro) comprising six beneficial proline substitutions was tried, and positive results were obtained [37,96] (Figure 2B,C). The higher yield and enhanced stability of HexaPro than the S-2P construct demonstrated a promising antigen design, and some vaccine candidates are being developed [87,97].

Besides proline mutation, Trimer-Tag was another choice for keeping the native-like prefusion form of trimeric spike proteins [71,98] (Figure 2D). Specifically, the human C-propeptide of α1(I) collagen (Trimer-Tag) was capable of self-trimerization via disulfide bonds, thus forming a disulfide bond-linked homotrimer. By fusing Trimer-Tag to the C-terminus of the ectodomain of wild-type SARS-CoV-2 S protein, the vaccine candidate, S-Trimer, was prepared. S-trimer keeps the crucial antigenic epitopes necessary for viral neutralization, and this construct represents a universal stabilization approach for other trimeric antigens. In phase 2 and 3 trials, two doses of the S-Trimer vaccine plus CpG and alum showed significant protection against circulating SAR-CoV-2 viruses, including the delta variant [15].

As immunodominant epitopes, RBD accounts for 90% of serum neutralizing activity [58,99] and is an attractive antigen target besides the spike protein mentioned above. Similarly, structure-guided vaccine design was involved. The receptor-binding motif (RBM) is an important recognition site of nAbs and interacts with ACE2 directly. The crystal structure reveals the RBD-dimer fully exposes dual RBMs [100] (Figure 2E); this inspires a structure-based design that the tandem repeat single-chain dimer may achieve higher vaccine efficacy than the conventional monomeric form [101]. Up to now, disulfide-linked dimer [102], interferon (IFN)-armed dimer [72], flexible pentapeptide (GGGGS)-engineered dimeric RBD [103], etc. are developed, and these vaccines appear safe and elicit strong antiviral immune responses. In particular, a COVID-19 vaccine candidate, ZF2001 (disulfide-linked RBD dimer), has been authorized for emergency use in China [30] (Figure 2F).

## 4. T-Cell-Based Vaccines

**Figure 3:**
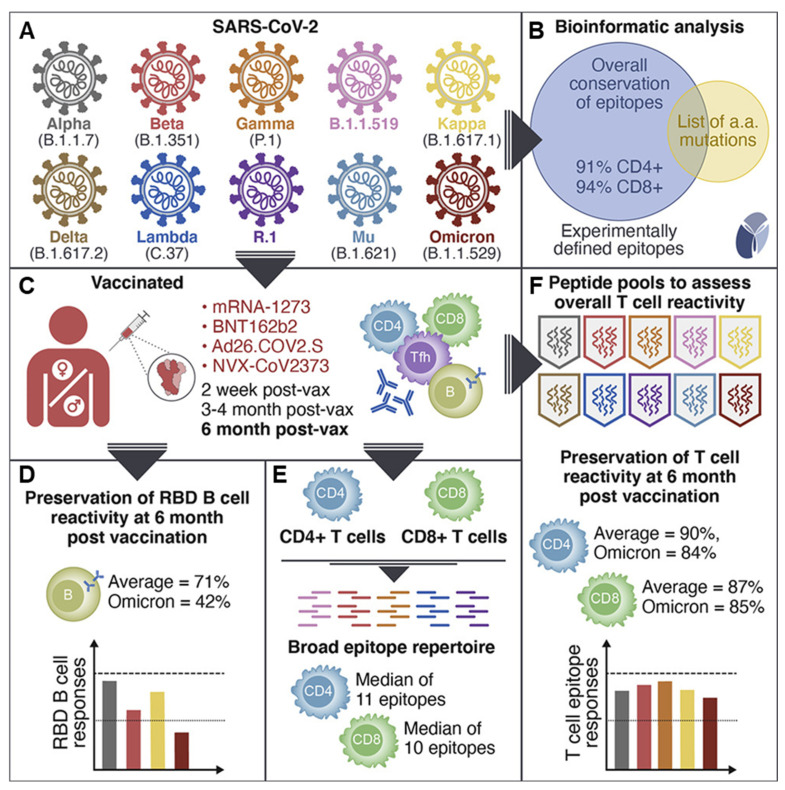
SARS-CoV-2 vaccination induces robust T-cell responses. Compared with decreased B cell responses, T-cell responses are preserved across different vaccine platforms. (**A**) SARS-CoV-2 and emerging variants. (**B**) Prediction of conserved T-cell epitopes. 91% and 94% of CD4^+^ and CD8^+^ T-cell epitopes are conserved on average, respectively. (**C**) T-cell responses in donors vaccinated with mRNA-1273, BNT162b2, Ad26.COV2.S, or NVX-CoV2373. (**D**) 71% memory B cells against variants on average are observed, and 42% for Omicron. (**E**) T-cell epitope repertoire analysis revealed a median of 11 and 10 spike epitopes recognized by CD4^+^ and CD8^+^ T cells. (**F**) 90% CD4^+^ (84% against Omicron) and 87% CD8^+^ (85% against Omicron) of memory T-cell responses were preserved against variants on average. Reproduced with permission from Ref. [110]. Copyright 2022, Elsevier.

Traditionally, antiviral vaccines rely on the induction of nAbs, and the efficacy of vaccines is dependent on the monitoring of sera antibody titers. Humoral immunity alone is insufficient to end the current COVID-19 pandemic and prevent a recurrence, owing to the genetic evolution and short-lived antibodies [104,105]. Recent findings point out that cross-reactive T-cell responses, especially tissue-resident memory T cells in the respiratory tract, could provide efficient heterologous immunity for respiratory infections [106,107,108]. T-cell-base immune response has been engaged to develop robust and long-lasting protective vaccines for SARS-CoV-2 [73,109,110] (Figure 3), and a combination of both humoral and cellular immunity might presumably be the most effective strategy. T cells have been recognized as a key element for tumor immunotherapy, and various works of research have leveraged cytotoxic T cells to attack tumors, but for antivirus treatment, associations between viruses and T cells are lacking; more attention should be paid to the functions of T cells.

To fulfill in silico prediction of immunogenic and conserved T-cell epitopes, some computational tools have been developed [111,112], thus providing precise guidance for antigen selection. Briefly, T-cell epitopes are determined by the specific algorithm and validated by experiments. Notably, the identification of mutationally constrained cytotoxic T lymphocytes (CTLs) epitopes in people with diverse HLA alleles is extensive, where the whole proteome of SARS-CoV-2 is defined [38]. What is more, some studies have advanced to elucidate multiple protective mechanisms of current administrated vaccines, and critical roles of T cells have been renewed [113,114,115,116]. However, the knowledge about T-cell-based immunity for virus control is limited so far. The relationship between nAbs and T-cell immune response, whether the magnitude of T-cell immune response is enough for durable efficacy, and how to tune the T-cell repertoire approximately, etc., are largely undetermined. Collectively, to fully leverage both nAbs and CTLs for next-generation vaccine design, much remains to be dissected.

## 5. Respiratory Mucosal Delivery

**Figure 4:**
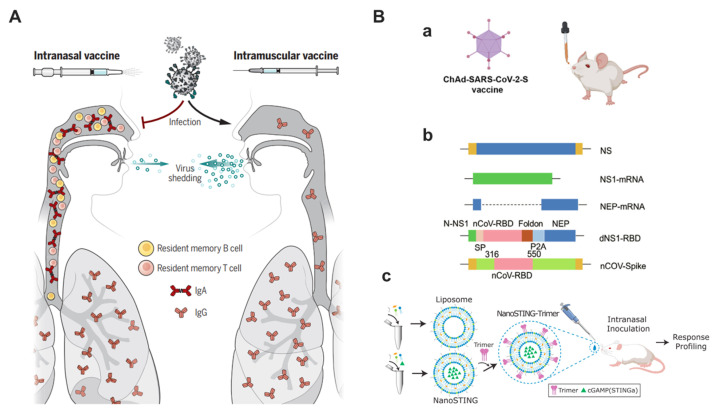
Respiratory mucosal delivery. (**A**) Intranasal vaccination can efficiently induce serum IgG, mucosal IgA, and tissue-resident cellular responses, thus potentially providing sterilizing immunity in the upper respiratory tract. Reproduced with permission from Ref. [120]. Copyright 2021, AAAS. (**B**) Representative mucosal-delivered vaccine candidates. (a) Adenoviral vaccine encoding stabilized spike protein. Copyright 2020, Elsevier. (b) Live attenuated influenza vaccine-based SARS-CoV-2 vaccine. Nonstructural protein 1, NS1. Deletion of NS1, dNS1. Copyright 2022, Elsevier. (c) An intranasal subunit vaccine. Copyright 2021, Elsevier. Reproduced with permission from Refs. [33,42,122].

The respiratory tract is the first line of defense against SARS-CoV-2, and intramuscularly injected vaccines are poor at controlling viral replication and nasal shedding in the upper respiratory tract [117]. However, intranasal vaccination could trigger robust protective immune responses at the initial invasion site of infection [118], showing the remarkable capacity to destroy the life cycle of respiratory pathogens. Besides non-invasive needle-free delivery, intranasal vaccination possesses a superior advantage in eliciting sterilizing immunity in the upper airway rather than only the lower respiratory tract, thus not only offering defense against symptomatic diseases but also preventing asymptomatic transmissions [119,120] (Figure 4A).

Mucosal immunity is a complex framework. The respiratory epithelial layer, innate immunity, and adaptive mucosal immunity constitute the defense lines against mucosal pathogens. Among these, mucosal secretory IgA (sIgA) antibodies and resident memory T (T_RM_) cells are vital for adaptive immune responses [121]. To develop fully competent mucosal vaccines, applicable antigens, mucosal adjuvants, and delivery vesicles should be comprehensively considered to reach fine-tuned formulations.

Learning from the licensed mucosal vaccines in past decades, some mucosal-delivered vaccine candidates for SARS-CoV-2 have been developed, including virus-vectored vaccines [43,76,122], live attenuated vaccines [32,33,123], and intranasal subunit vaccines [42], etc. (Figure 4B). Whereas the adenovirus is a mature and easily modified system, Ad viral vector vaccines are highly concerned, the inserted gene can express spike proteins in vivo and then the specific immune responses are activated. For influenza-based vaccines, the influenza viruses encoding RBD of spike protein are conditionally replicated (cold-adaptive, preferred for 33 °C), which means attenuated in humans. Subunit vaccines are usually administered via the intramuscular route, intranasal injection may take advantage of inducing mucosal immunity in the nasal compartment, which is desirable for defending against SARS-CoV-2. Notably, an orally administered aerosolized Ad5-nCoV vaccine has just been licensed for emergency use as a sequential booster in China (Clinical trial NCT05043259). The orally administered aerosolized Ad5-nCoV was confirmed safe and highly immunogenic after two-dose priming with CoronaVac (an approved inactivated COVID-19 vaccine) in clinical studies [124]. This would bolster the global efforts in developing respiratory mucosal-delivered COVID-19 vaccines.

## 6. Nanotechnologies

**Figure 5:**
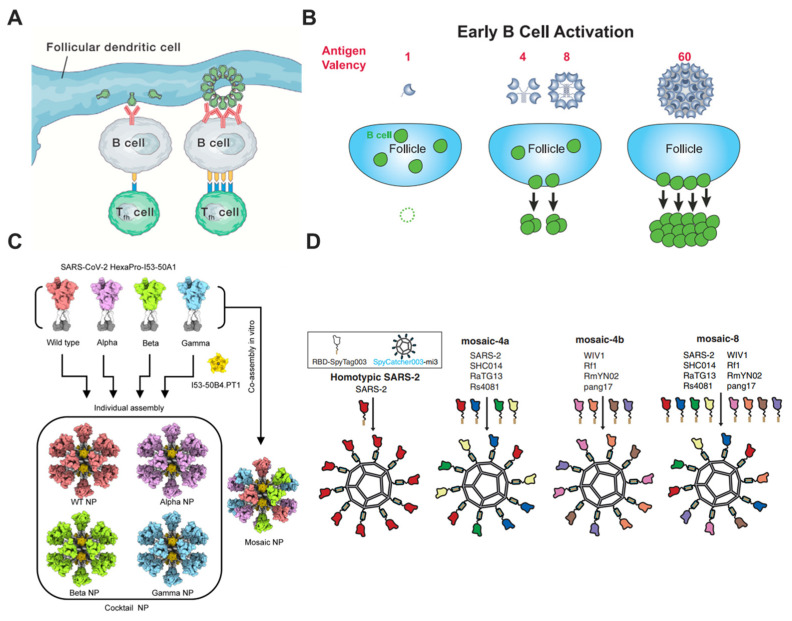
Nanoparticle vaccines. Nanoparticles can drain into lymph nodes, and their unique physical properties (size, shape, charge, etc.) are beneficial for antigen presentation while interacting with resident DCs. (**A**) Nanoparticles could engage stronger interactions between antigen and B cell and between B cell and the follicular helper T-cell (Tfh), resulting in stronger immunity. Reproduced with permission from Ref. [125]. Copyright 2019, Elsevier. (**B**) High valency enables robust activation and effector differentiation of B cells. Reproduced with permission from Ref. [129]. Copyright 2020, Elsevier. (**C**) Schematic diagram of SARS-COV-2 HexaPro bearing nanoparticle vaccines. Different antigens were genetically fused to I53-50A1, and an excess pentameric I53-50B.4PT1 was added at an equimolar ratio for co-assembly in vitro. Reproduced with permission from Ref. [78]. Copyright 2022, Springer Nature. (**D**) Mosaic nanoparticles were constructed by the SpyCatcher-SpyTag system. SpyCatcher is derived from a *Streptococcus pyogenes* surface protein, which can bind the 13-amino-acid peptide SpyTag. This system is widely applicated for protein ligation. Reproduced with permission from Ref. [35]. Copyright 2021, AAAS.

The thriving nanotechnologies-based platforms provide an appealing option for COVID-19 vaccine preparation [125]. Owing to their good immunogenicity, stable structure and strong ability to display foreign proteins, nanoparticle vaccines attracted broad interest in recent years. In fact, virus-like particles (VLPs) are a type of natural nanoparticle platform which have been broadly employed commercially for vaccine manufacturing [126], such as hepatitis B virus (HBV) [127] and human papillomavirus (HPV) [128]. Self-assembled by viral proteins without genetic materials, VLPs possess nanoscale size and regular structure, which are well-arranged mimics of original viruses. Furthermore, nanoarchitectures with modular nanocages for the display of antigens have distinct advantages: enrichment of antigens (enhanced antigen valency) [129,130], multivalent display of antigens (broadly protective responses against mutated strains) [131,132] (Figure 5A,B). As mimics of nano-scaled viruses, nanoparticle vaccines can serve as a rational platform for vaccine development.

Through rational or computational design, lots of self-assembly nanoparticle vaccines with antigens optimization have been developed to combat the SARS-CoV-2 pandemic, and some inspiring results have been reported in preclinical and clinical studies [133,134]. Among various formats, mosaic nanoparticle vaccines have become the research focus and take the potential to revolutionize the field of vaccine development [35,78,135]. Scaffolded by self-assembled protein nanoparticle platforms, such as I53-50 [78] and SpyCatcher003 [35,135], etc., distinct spike/RBD antigens from the SARS-CoV-2 prototype and its variants could be co-displayed in the tailored mosaic nanoparticles (Figure 5C,D). Mosaic nanoparticle vaccines could elicit cross-reactive immune responses, demonstrating broad protection potential for SARS-CoV-2, as well as current and future emerging variants. And notably, both matched and mismatched viral challenges could be protected by mosaic nanoparticle vaccines [135]. Although nanoparticle vaccines have shown brilliant prospects, there are still some concerns that should not be ignored. Firstly, the design of nanoparticle vaccines has not yet reached the most cost-effective design scheme, and their biological distribution and metabolic clearance need to be further studied. Secondly, how to achieve large-scale manufacturing, sustainability and reproducibility of nanoparticle vaccines remain to be solved. Last but not the least, more research is urged to elucidate the interactions between nanoparticles and the body’s immune system. Altogether, further studies are expected to verify the safety and efficacy of COVID-19 mosaic nanoparticle vaccines in the real world.

## 7. Challenges and Future Perspective

Despite tremendous efforts that have been devoted to providing highly-effective vaccines against SARS-CoV-2 and emerging variants, there are many challenges to be solved to halt the pandemic. Firstly, the frequent mutations bring immune-escaping and breakthrough infection. Though various vaccine candidates, as discussed above, demonstrated distinguished protection effects in preclinical and clinical studies, some breakthrough infections have been reported [136,137]. Evolving variants have a profound uncertainty on the efficacy of currently available vaccines, which warrants further study and updated vaccines are called for. Moreover, the identification of conserved epitopes may be a good choice. Secondly, how long the immune responses post-vaccination will maintain remains to be explored [138,139]. Once waning immunity occurs, rapid transmission and asymptomatic spread of COVID-19 may pose a high risk to global public health. In this case, heterologous prime-boost vaccination may extend the duration of protection [140,141,142]. More follow-up studies are needed to determine the longevity of immunity. Thirdly, the majority of vaccines are thermo-sensitive; they rely on cold chain storage and transportation, which makes vaccine delivery complicated. To achieve the equitable distribution and convenient management of vaccines, thermo-stable vaccines are preferred. Lastly, individual differences, including gender, age, etc., may affect vaccine-induced immunity [143,144]. Associations incorporated require further investigation and understanding of these factors offer opportunities for the rational design of next-generation vaccine candidates.

Besides, novel and affordable adjuvants with optimal efficacy and safety profiles are urged for COVID-19 vaccines. Only a few adjuvants have been licensed for clinical usage in the pandemic, such as aluminum salts, Matrix-M, etc. [6,95]. Generally, adjuvants can enhance immune responses and reach antigen dose-sparing. Screening and selecting proper adjuvants for COVID-19 vaccines will enable more potent vaccine formulations.

## 8. Conclusions

Benefiting from the interdisciplinary studies of molecular biology, structural biology, bioinformatics, and materials science, to date, various kinds of vaccines have been approved to combat COVID-19, and the results from clinical trials are encouraging. In the past three years, clinically approved vaccines have demonstrated powerful effects in reducing severe COVID-19 and mortality. Nonetheless, effective vaccines are still in shortage worldwide to stem the ongoing SARS-CoV-2 pandemic and drive the world to return to pre-pandemic normalcy. We should move forward without hesitancy to address continuing challenges at the phase that the next round of global pandemic may arrive, and insights into novel vaccine design are necessary. In summary, this review provided detailed vaccine design insights into the COVID-19 pandemic to advance the development of desired vaccines with safety and long-term efficacy.

## Figures and Tables

**Table 1:** Novel vaccine design approaches utilized for SARS-CoV-2 and its variants.

Approaches	Principles	Examples
Structure-guided vaccine design	The metastable prefusion conformation is the functional state for spike proteins, stabilizing the prefusion form could preserve neutralization-sensitive epitopes.	S-2P [37,68,69], S-6P [70], S-trimer [15,71], RBD dimer [30,31,72], etc.
T-cell-based vaccines	Circulating antibodies may be short-lived, or of low magnitude and/or potency, T cells have an important role for COVID-19 outcome and maintenance of SARS-CoV-2 immunity.	Peptide vaccine [73], MVA-S [74,75], etc.
Respiratory mucosal delivery	To elicit mucosal IgA and T-cell mediated immune responses in the respiratory tract.	CA4-dNS1-nCoV-RBD [32,33], Ad-vectored [43,76], intranasal subunit vaccine [42], etc.
Nanotechnologies	Multivalent display of antigen enhances B-cell responses and can provide longer-lasting immunity than monovalent antigens.	Mosaic nanoparticles [35,77,78], Ferritin-based nanoparticles [79,80], etc.

**Table 2:** Comparison of the four summarized vaccine design strategies.

Approaches	Advantages	Limits
Structure-guided vaccine design	The immunogenicity and yield of target antigens are improved	The high-resolution structures are required
T-cell-based vaccines	Stronger CD8^+^ T-cell responses are provided	T-cell epitopes need to be identified
Respiratory mucosal delivery	Mucosal immunity in the respiratory tract can be elicited rapidly	Serological antibody titers are relatively lower than intramuscular injection
Nanotechnologies	Multivalent antigens can be displayed in one particle	The nanotoxicity needs further clinical investigation

## Data Availability

Not applicable.
